# Supplementary figures and images for: Terpenoid Biosynthesis Dominates among Secondary Metabolite Clusters in Mucoromycotina Genomes
Source: J Fungi (Basel). 2021 Apr 9;7(4):285. doi: 10.3390/jof7040285 (PMC8070225; doi:10.3390/jof7040285)

NRPS-like

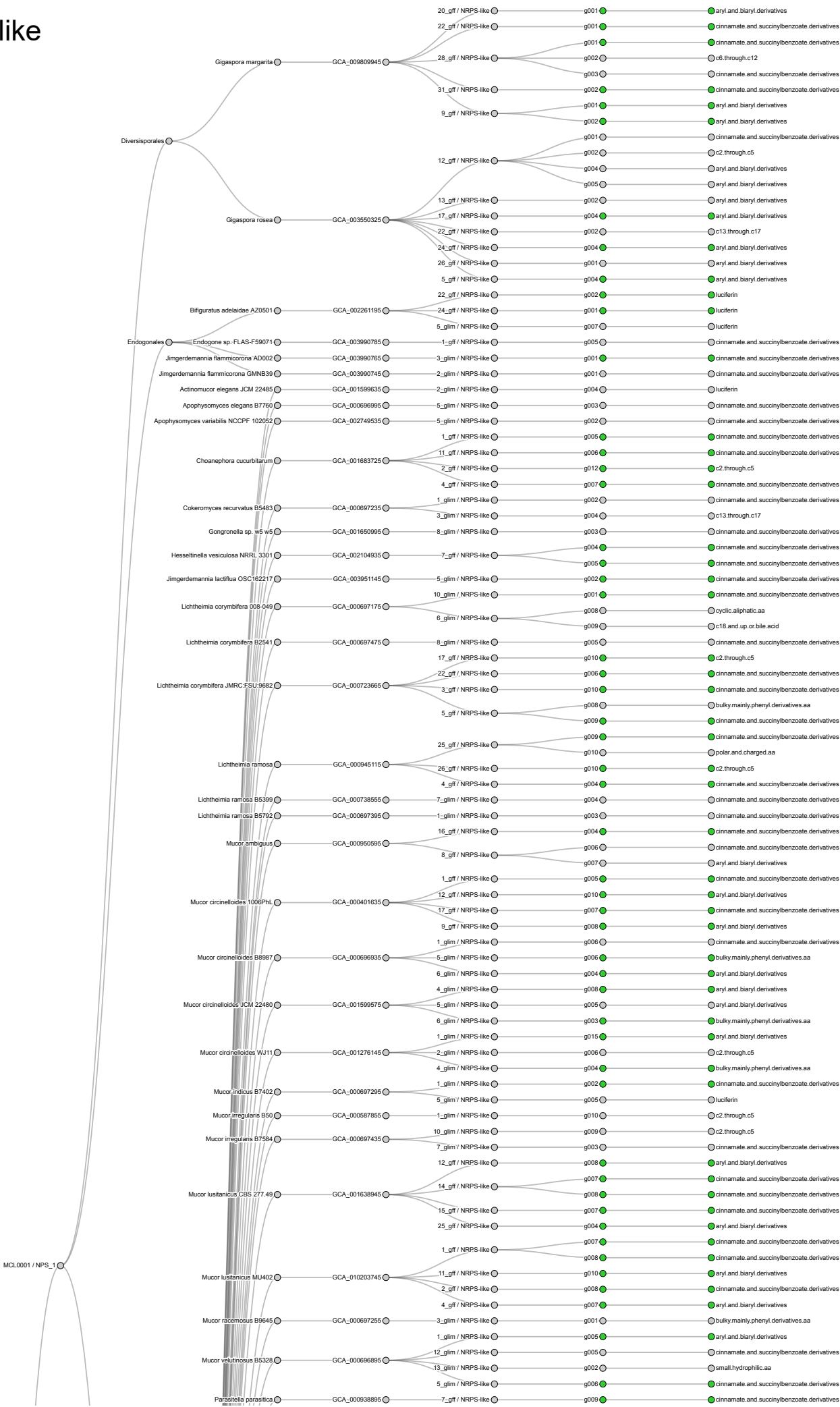

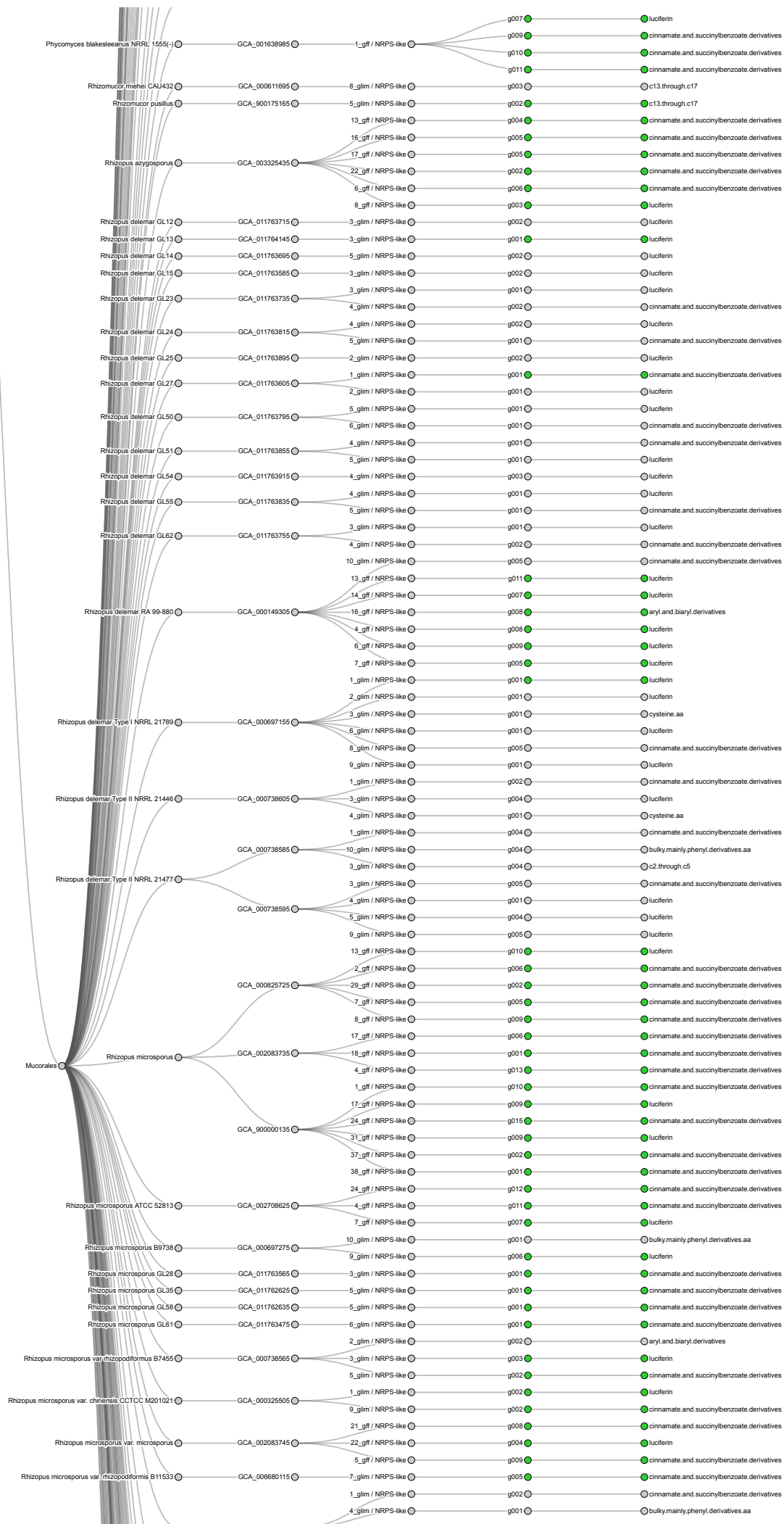

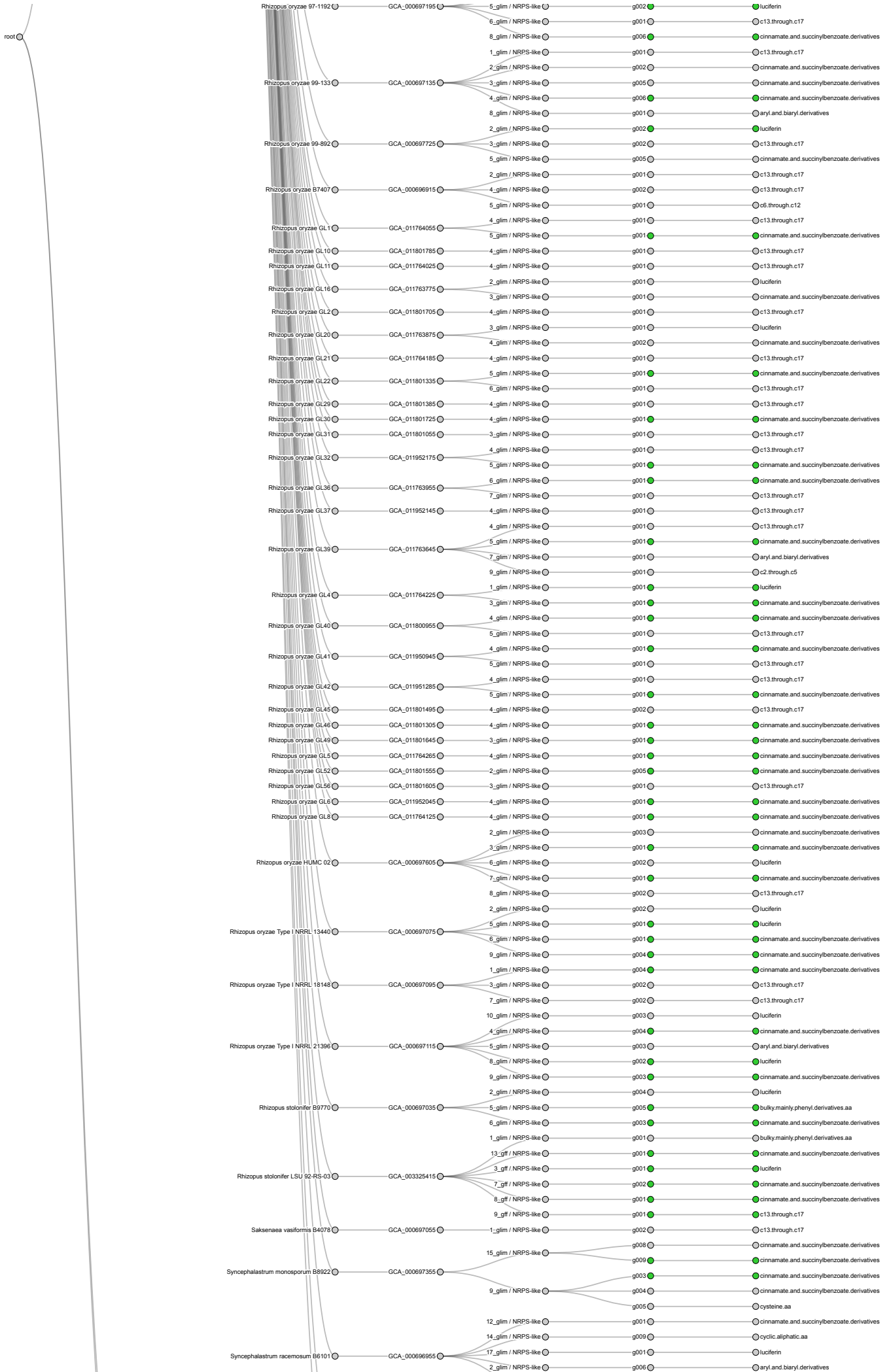

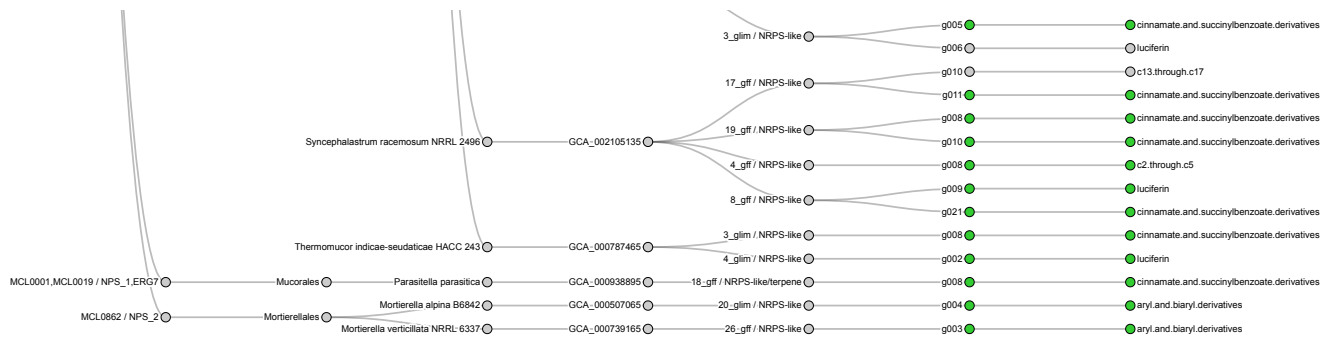

## NRPS

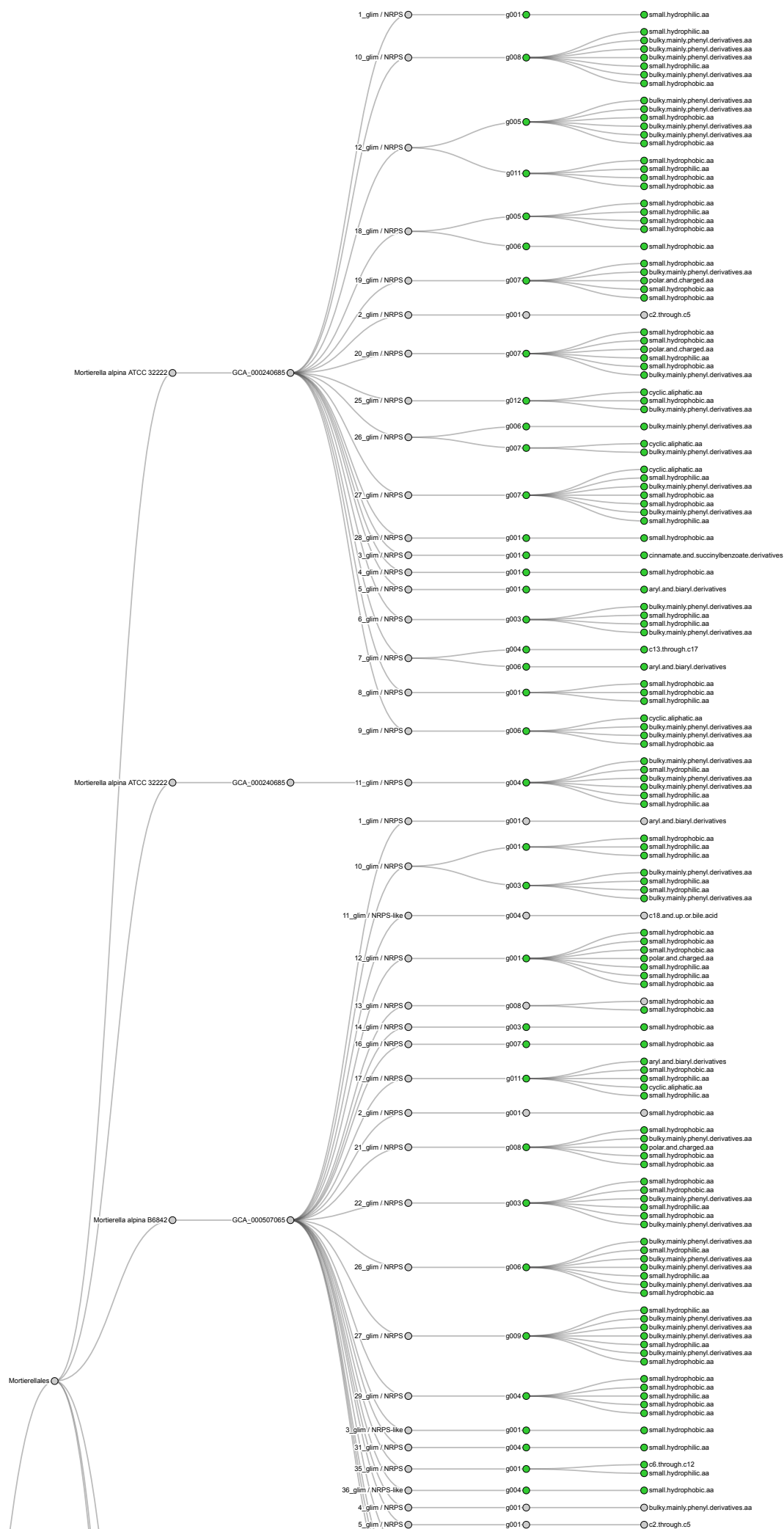

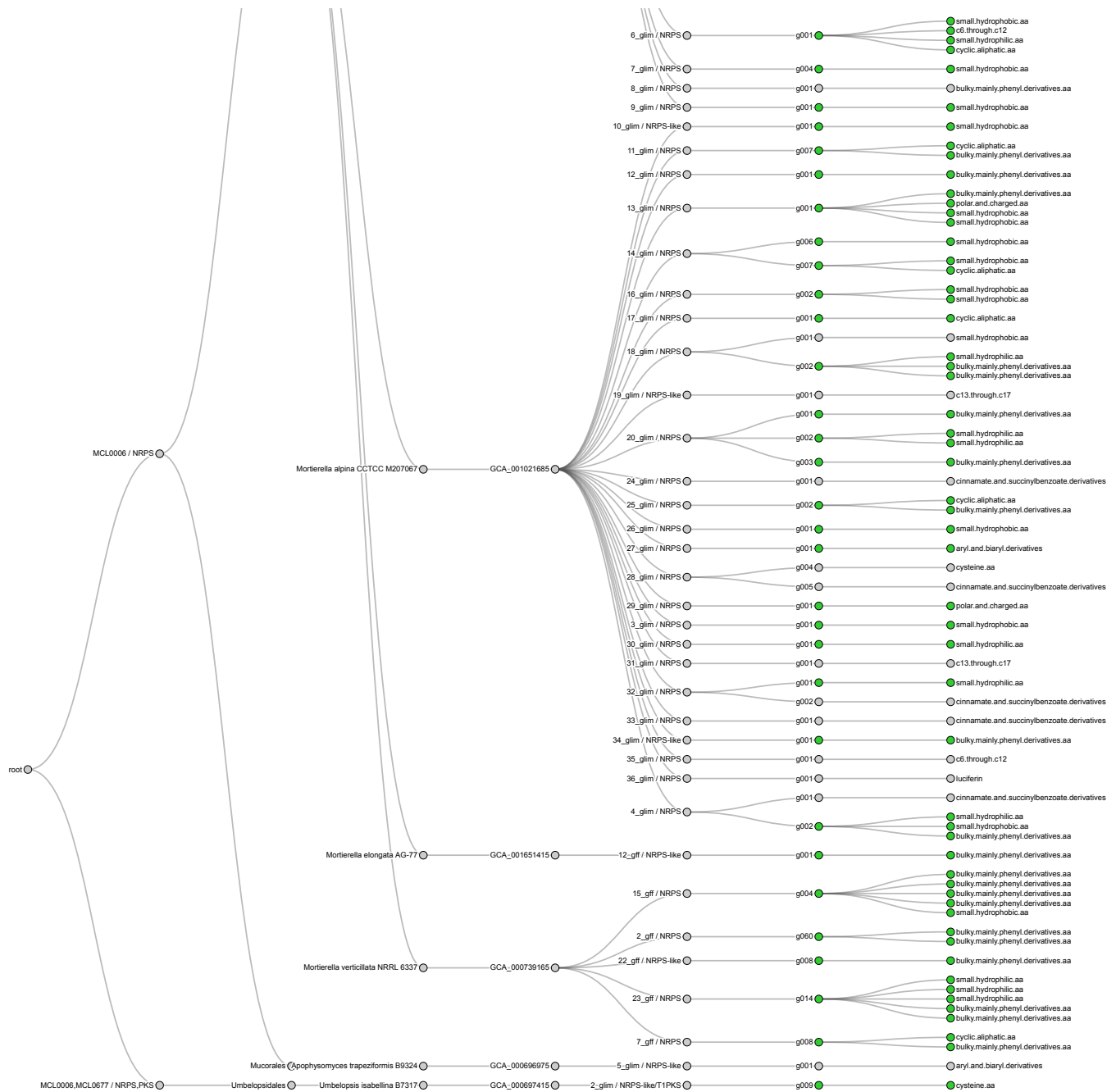

LYS2

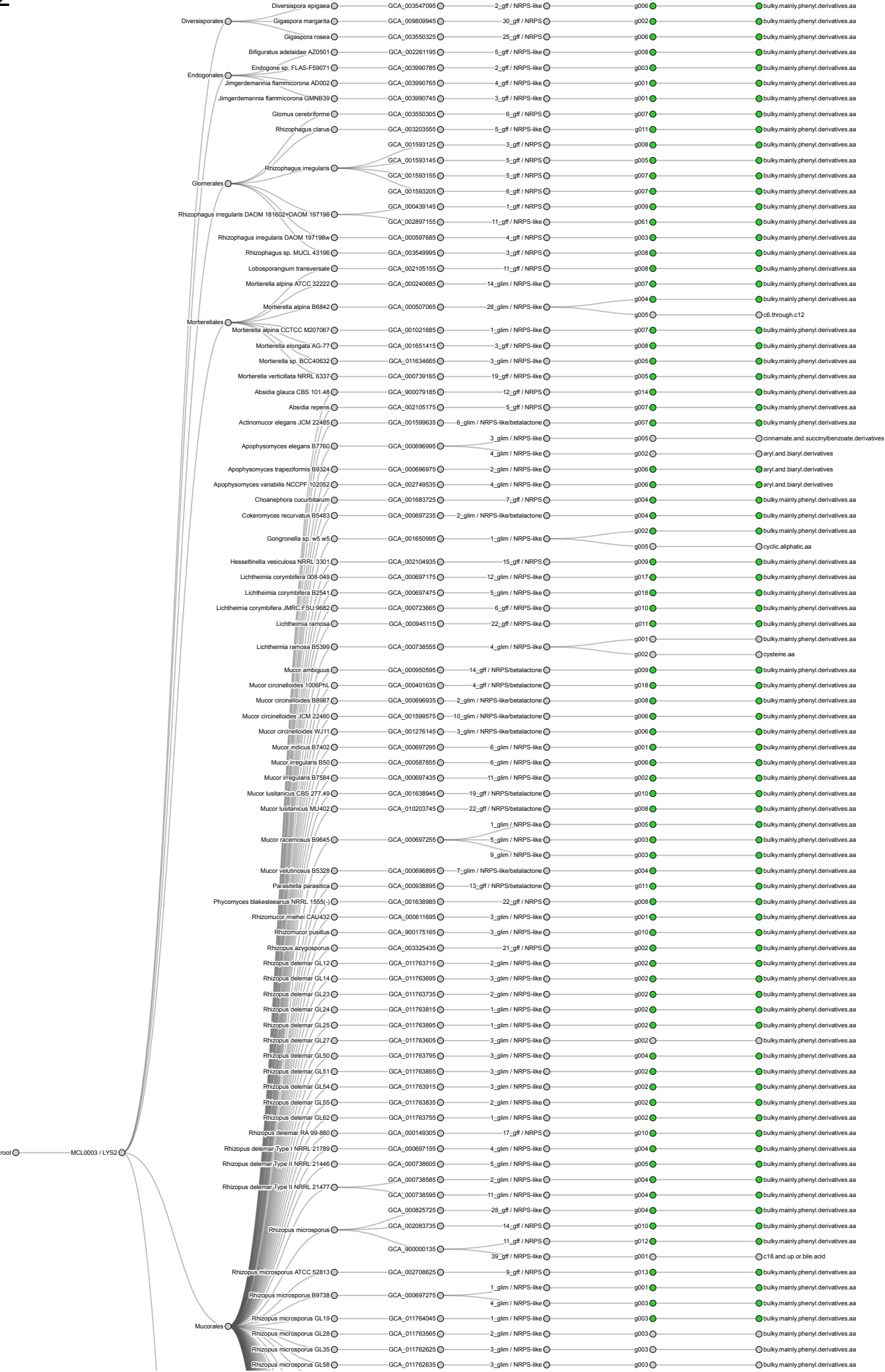

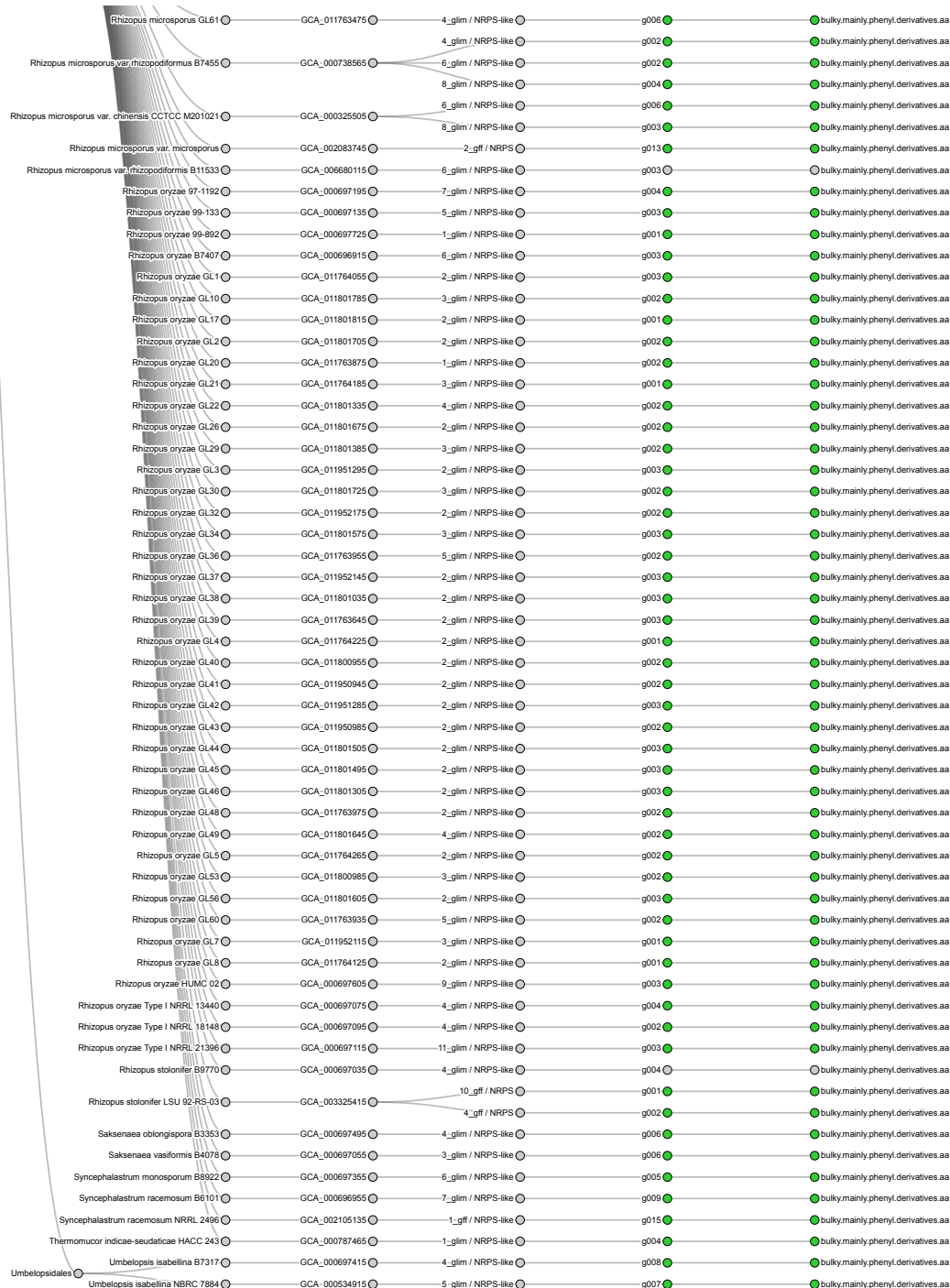

NRPS-PKS

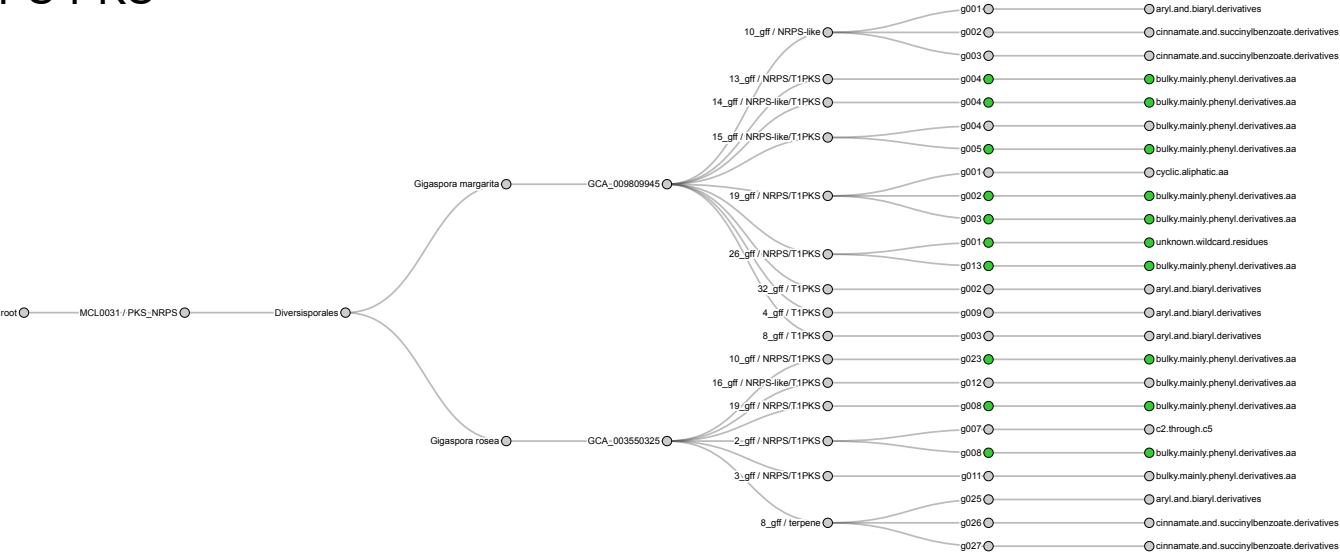

Supplement: Supplementary file 1 [file jof-07-00285-s001.zip › supplementary files/Supplementary Figure SF2.pdf]

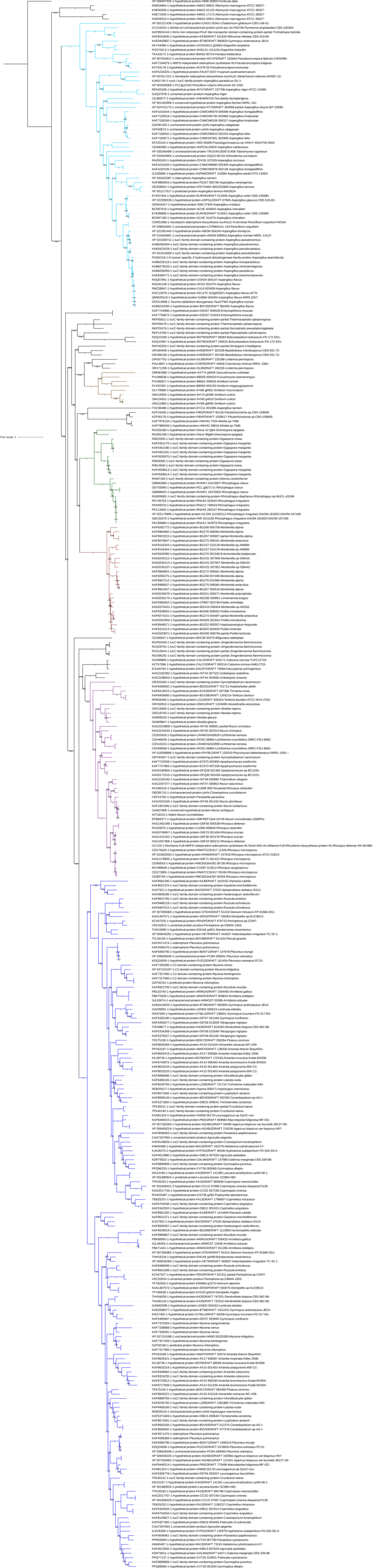

Supplement: Supplementary file 1 [file jof-07-00285-s001.zip › supplementary files/Supplementary Figure SF3.pdf]

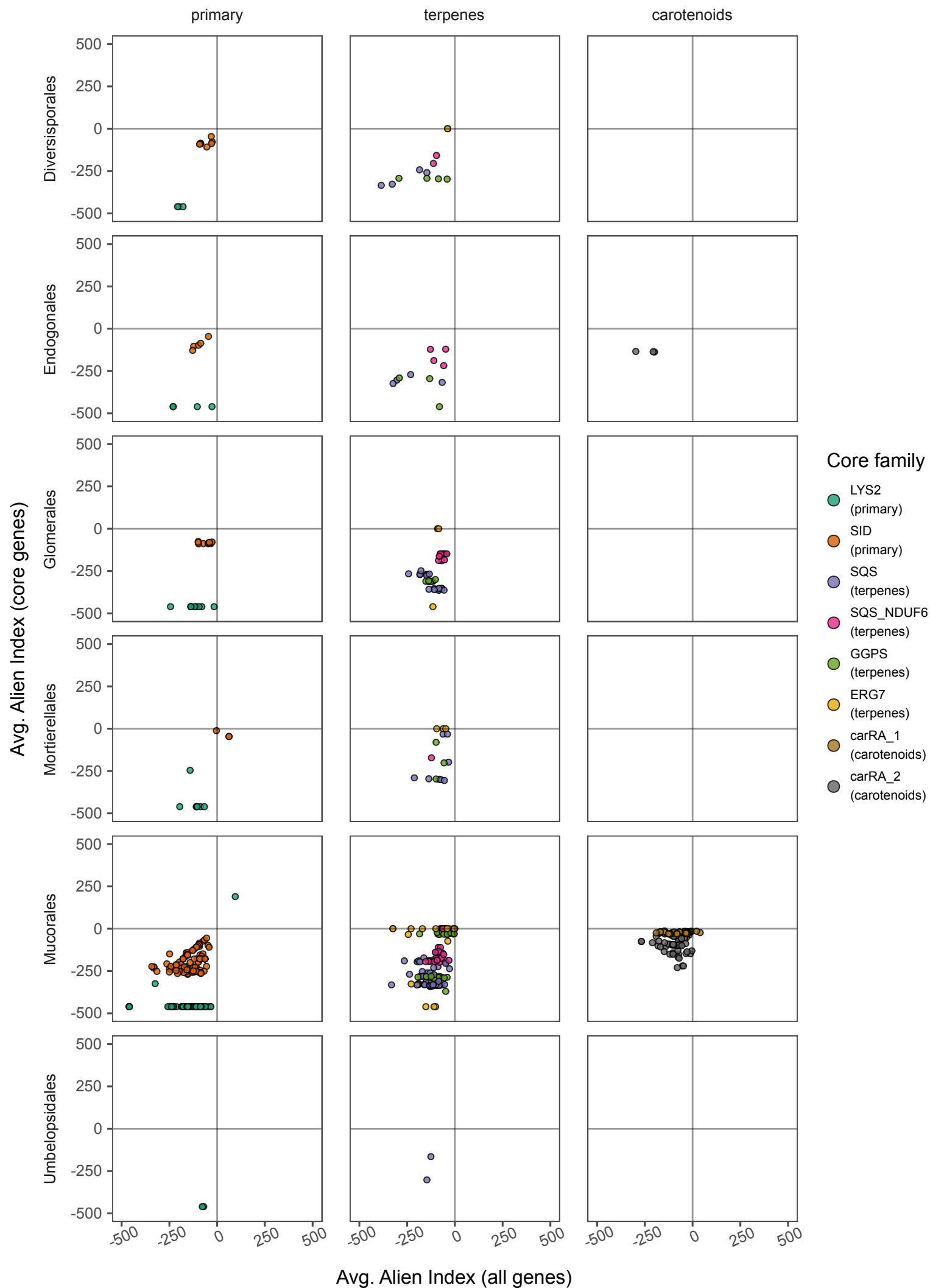

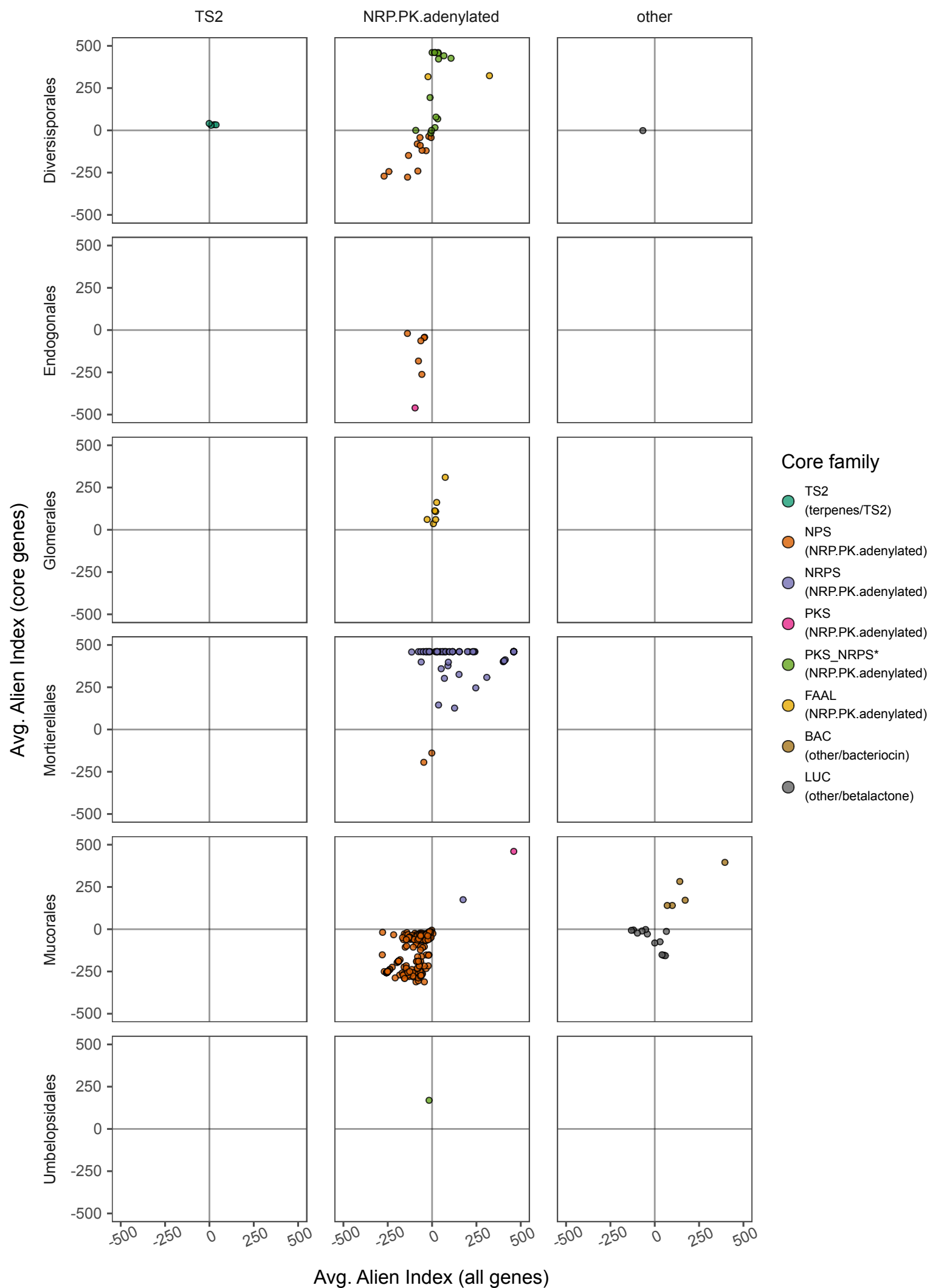

Supplement: Supplementary file 1 [file jof-07-00285-s001.zip › supplementary files/SupplementaryFigureSF1.pdf]
